# Glycyrrhizin Exerts Antioxidative Effects in H5N1 Influenza A Virus-Infected Cells and Inhibits Virus Replication and Pro-Inflammatory Gene Expression

**DOI:** 10.1371/journal.pone.0019705

**Published:** 2011-05-17

**Authors:** Martin Michaelis, Janina Geiler, Patrizia Naczk, Patchima Sithisarn, Anke Leutz, Hans Wilhelm Doerr, Jindrich Cinatl

**Affiliations:** Institut für Medizinische Virologie, Klinikum der J.W. Goethe-Universität, Frankfurt am Main, Germany; Johns Hopkins University - Bloomberg School of Public Health, United States of America

## Abstract

Glycyrrhizin is known to exert antiviral and anti-inflammatory effects. Here, the effects of an approved parenteral glycyrrhizin preparation (Stronger Neo-Minophafen C) were investigated on highly pathogenic influenza A H5N1 virus replication, H5N1-induced apoptosis, and H5N1-induced pro-inflammatory responses in lung epithelial (A549) cells. Therapeutic glycyrrhizin concentrations substantially inhibited H5N1-induced expression of the pro-inflammatory molecules CXCL10, interleukin 6, CCL2, and CCL5 (effective glycyrrhizin concentrations 25 to 50 µg/ml) but interfered with H5N1 replication and H5N1-induced apoptosis to a lesser extent (effective glycyrrhizin concentrations 100 µg/ml or higher). Glycyrrhizin also diminished monocyte migration towards supernatants of H5N1-infected A549 cells. The mechanism by which glycyrrhizin interferes with H5N1 replication and H5N1-induced pro-inflammatory gene expression includes inhibition of H5N1-induced formation of reactive oxygen species and (in turn) reduced activation of NFκB, JNK, and p38, redox-sensitive signalling events known to be relevant for influenza A virus replication. Therefore, glycyrrhizin may complement the arsenal of potential drugs for the treatment of H5N1 disease.

## Introduction

Highly pathogenic H5N1 influenza A viruses are considered to be potential influenza pandemic progenitors [1]–[6]. At least for the first wave of an H5N1 pandemic, no sufficient amounts of adequate vaccines will be available [1]–[4], [6]–[8]. Therefore, antiviral therapy for influenza A viruses including highly pathogenic H5N1 virus strains remains of great importance for the first line defense against the virus [1]–[4], [6], [9].

The neuraminidase inhibitors oseltamivir and zanamivir as well as the adamantanes amantadin and rimantadin that interfere with the influenza M2 protein are licensed for the treament of influenza [1]–[4], [6]. However, the use of both drug classes is limited by the emergence of resistant virus strains. In seasonal influenza strains, the majority of H3N2 viruses and a great proportion of H1N1 viruses in humans are now considered to be amantadine- and rimantadine-resistant [10]–[13]. Moreover, a drastic increase in oseltamivir-resistant H1N1 viruses has been reported during the 2007/2008 influenza season in the northern hemisphere [14]–[17]. Preliminary data from the United States predict a further rise for the 2008/2009 season, possibly resulting in more than 90% of the circulating H1N1 strains to be oseltamivir resistant [14].

H5N1 virus strains appear to be generally less sensitive to antiviral treatment than seasonal influenza A virus strains and treatment-resistant H5N1 strains emerge [1]–[4], [6], [18]–[21]. Moreover, parenteral agents for the treatment of seriously ill patients are missing. Glycyrrhizin, a triterpene saponine, is a constituent of licorice root. It has been found to interfere with replication and/or cytopathogenic effect (CPE) induction of many viruses including respiratory viruses such as respiratory syncytial virus, SARS coronavirus, HIV, and influenza viruses [22]–[28]. Moreover, anti-inflammatory and immunomodulatory properties were attributed to glycyrrhizin [26]. The severity of human H5N1 disease has been associated with hypercytokinaemia (“cytokine storm”) [29], [30]. Delayed antiviral plus immunomodulator treatment reduced H5N1-induced mortality in mice [31]. Therefore, anti-inflammatory and immunomodulatory effects exerted by glycyrrhizin may be beneficial for treatment of H5N1. Also, glycyrrhizin is a known antioxidant [26] and antioxidants were already shown to interfere with influenza A virus replication and virus-induced pro-inflammatory responses [32]–[34].

Stronger Neo-Minophagen C (SNMC) is a glycyrrhizin preparation (available as tablets or parenteral formulation) that is approved in Japan for the treatment of chronic hepatic diseases and is marketed in Japan, China, Korea, Taiwan, Indonesia, India, and Mongolia. Here, we investigated the influence of SNMC on H5N1 replication, on H5N1-induced cytokine expression, on H5N1-induced cellular oxidative stress, and on critical H5N1-induced cellular signalling events in human pneumocytes (A549 cell line).

## Materials and Methods

### Drugs

Glycyrrhizin (Stronger Neo Minophagen C) was obtained from Minophagen Pharmaceuticals Co., Ltd. (Tokyo, Japan).

### Virus strains

The influenza strain A/Vietnam/1203/04 (H5N1) was received from the WHO Influenza Centre (National Institute for Medical Research, London, UK). The H5N1 influenza strain A/Thailand/1(Kan-1)/04 was obtained from Prof. Pilaipan Puthavathana (Mahidol University, Bangkok, Thailand).

Virus stocks were prepared by infecting Vero cells (African green monkey kidney; ATCC, Manassas, VA) and aliquots were stored at −80°C. Virus titres were determined as 50% tissue culture infectious dose (TCID_50_/ml) in confluent Vero cells in 96-well microtiter plates.

### Cells

A549 cells (human lung carcinoma; ATCC: CCL-185, obtained from LGC Standards GmbH, Wesel, Germany) were grown at 37°C in minimal essential medium (MEM) supplemented with 10% FBS, 100 IU/ml of penicillin and 100 µg/ml streptomycin.

Human monocytes were isolated from buffy coats of healthy donors, obtained from Institute of Transfusion Medicine and Immune Haematology, German Red Cross Blood Donor Center, Johann Wolfgang Goethe-University, Frankfurt am Main. After centrifugation on Ficoll (Biocoll)-Hypaque density gradient (Biochrom AG, Berlin, Germany), mononuclear cells were collected from the interface and washed with PBS. Then, monocytes were isolated using magnetically labeled CD14 MicroBeads (Miltenyi Biotec GmbH, Bergisch Gladbach, Germany) following the manufacturer's instructions. Monocytes were cultivated in IMDM supplemented with 10% pooled human serum, 100 IU/ml of penicillin, and 100 µg/ml streptomycin.

### Cell viability assay

The cellular viability was assessed on confluent cell layers with CellTiter-Glo® Luminescent Cell Viability Assay (Promega GmbH, Mannheim, Germany) according to the manufacturers' protocol. Cell viability was expressed as percentage of non-treated control.

### Detection of influenza A nucleoprotein

To determine intracellular NP localisation, H5N1-infected A549 were fixed 8 hours p.i. for 15 min with ice-cold acetone/methanol (40∶60, Mallinckrodt Baker B.V., Deventer, The Netherlands) and stained with a mouse monoclonal antibody (1 h incubation, 1∶1000 in PBS) directed against the influenza A virus nucleoprotein (NP) (Millipore, Molsheim, France). An Alexa Fluor 488 goat anti-mouse IgG (H&L) (Invitrogen, Eugene, Oregon, USA) was used (1 h incubation, 1∶1000 in PBS) as secondary antibody. Nuclei were stained using 4′,6-diamidino-2-phenylindole (DAPI) (Sigma-Aldrich Chemie GmbH, Munich, Germany). Fluorescence was visualised using Olympus IX 1 fluorescence microscope (Olympus, Planegg, Germany).

For flow cytometric analysis, the same antibodies were used.

### Cytopathogenic effect (CPE) reduction assay

The cytopathogenic effect (CPE) reduction assay was performed as described before [34]. Confluent A549 cell monolayers grown in 96-well microtitre plates were infected with influenza A strains at the indicated multiplicities of infection (MOIs). After a one hour adsorption period, cells were washed to remove non-detached virus. The virus-induced CPE was recorded at 24 h post infection (p.i.).

Unless otherwise stated, A549 cells were continuously treated with glycyrrhizin starting with a 1 h pre-incubation period. For time-of-addition experiments, glycyrrhizin was added exclusively during the 1 h pre-incubation period, exclusively during the 1 h adsorption period, or after exclusively after the wash-out of input virus.

### Real-time PCR

Total RNA was isolated from cell cultures using TRI reagent (Sigma-Aldrich, Munich, Germany). Real time PCR for H5 was performed using described methods [35]. The following primers were used: sense 5′ acg tat gac tac ccg cag tat tca g 3′; antisense 5′ aga cca gcy acc atg att gc 3′; probe 6-FAM-tca aca gtg gcg agt tcc cta gca-TAMRA.

### Sub-G1 cells

The fraction of cells with fractional DNA content (“sub-G1” cell subpopulation) indicates cytotoxicity. Sub-G1 cells are considered to be dead (usually apoptotic) cells. Cells were fixed with 70% ethanol for two hours at −20°C. The cellular DNA was stained using propidium iodide (20 µg/ml) and analysed by flow cytometry (FacsCalibur, BD Biosciences, Heidelberg, Germany).

### Caspase activation

Caspase activation was measured using the Caspase-Glo 8, 9, or 3/7 Assays (Promega, Mannheim, Germany) following the manufacturer's instructions.

### Cytokine/Chemokine secretion

Cell culture supernatants were collected and frozen at −80°C. Cytokines/chemokines were quantified by specific ELISA Duo Sets (R&D Systems GmbH, Wiesbaden, Germany) following the manufacturer's instructions.

### Nuclear factor κB (NFκB) activity

NFκB activity was investigated in H5N1 (MOI 0.01)-infected cells by quantification of the NFκB subunits Rel A (p65) and NFκB1 (p50) from nuclear extracts using the TransAM™ transcription factor DNA-binding ELISAs (Active Motif, Rixensart, Belgium). Nuclear extract were prepared using the Nuclear Extract Kit (Active Motif, Carlsbad, CA, USA) following the manufacturer's instruction.

### Migration assay

Cell culture supernatants were investigated for chemotactic activity by measurement of the activity to induce monocyte migration through membrane inserts in 24-well plates (pore size 8 µm; BD Biosciences, Heidelberg, Germany). Monocytes (1×10^6^ in 100 µl of IMDM with 10% pooled human serum) were added into the cell culture inserts (upper chamber) and cell culture supernatants (300 µl), were added to the lower chamber of the well. After a 48 h incubation period, cells were fixed with 4% paraformaldehyde and permeabilised with PBS containing 0.3% Tritron X-100. Then, nuclei were stained with 4′,6-diamidino-2-phenylindole (DAPI). The upper side of the membrane was wiped with a wet swab to remove the cells, while the lower side of the membrane was rinsed with PBS. The number of cells at the lower side of each membrane was quantified by counting of cells from three randomly chosen sections (3.7 mm^2^) using an Olympus IX 1 fluorescence microscope (Olympus, Planegg, Germany).

### Western blot

Cells were lysed in Triton X-sample buffer and separated by SDS-PAGE. Nuclear extract were prepared using the Nuclear Extract Kit (Active Motif, Carlsbad, CA, USA) following the manufacturer's instruction. Proteins were detected using specific antibodies against β-actin (Sigma-Aldrich Chemie GmbH, Munich, Germany), JNK, phosphorylated JNK, p38, or phosphorylated p38, (all purchased from New England Biolabs GmbH, Frankfurt am Main, Germany) and were visualised by enhanced chemiluminescence using a commercially available kit (Amersham, Freiburg, Germany).

### Determination of cellular oxidative stress

Reactive oxygen species (ROS) were detected using the Image-iT LIVE Green Reactive Oxygen Species Kit (Molecular Probes, distributed by Invitrogen, Karlsruhe, Germany).

### Statistical analysis

Two groups were compared by t-test. More groups were compared by ANOVA with subsequent Student-Newman-Keuls test.

## Results

### Influence of glycyrrhizin on replication of H5N1 virus in A549 cells

**Figure 1:**
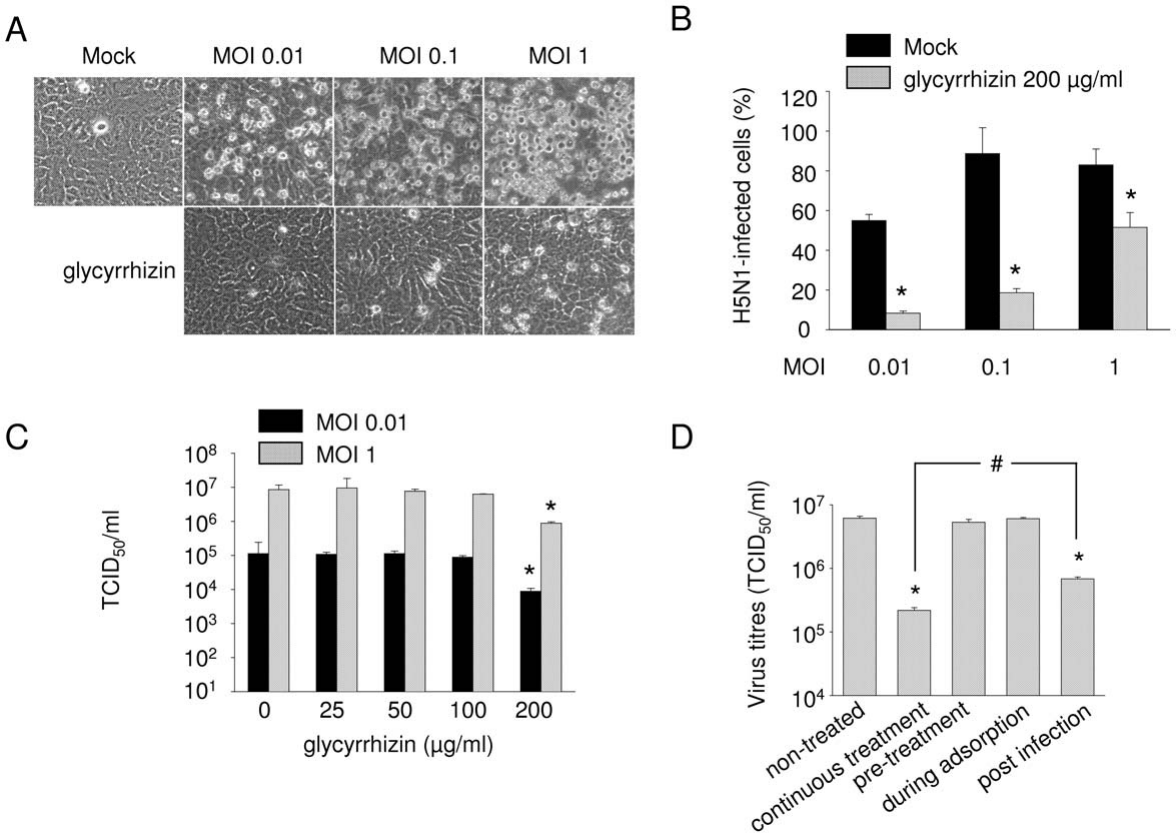
Influence of glycyrrhizin on H5N1 replication in A549 cells. A) Representative pictures of non-infected A549 cells (Mock) and cytopathogenic effect formation in A549 cells infected with H5N1 A/Thailand/1(Kan-1)/04 at different multiplicities of infection (MOI) without or with glycyrrhizin (200 µg/ml) treatment for 24 h. B) Influence of glycyrrhizin 200 µg/ml on the number of H5N1 A/Thailand/1(Kan-1)/04-infected A549 cells determined by flow cytometric analysis of nucleoprotein-positive cells 24 h post infection. C) Influence of different glycyrrhizin concentrations on viral titres determined in A549 cells infected with H5N1 A/Thailand/1(Kan-1)/04 (MOI 0.01 or MOI 1) 24 h post infection. D). Influence of different glycyrrhizin administration schedules on H5N1 A/Thailand/1(Kan-1)/04 (MOI 0.01) viral titres determined in A549 cells 24 h post infection. Cells were treated without glycyrrhizin (non-treated), with glycyrrhizin 200 µg/ml continuously starting with a 1 h pre-incubation period (continuous treatment), with glycyrrhizin 200 µg/ml solely for a 1 h pre-incubation period (pre-treatment), with glycyrrhizin 200 µg/ml solely during the 1 h adsorption period, or with glycyrrhizin 200 µg/ml added after virus adsorption. Pro-longed pre-treatment periods did not result in increased virus inhibitory effects of the continuous treatment regimen (not shown). * P<0.05 relative to non-treated virus control, # P<0.05.

The A549 cell line, derived from a human pulmonary adenocarcinoma, is an established model for type II pneumocytes [36], and commonly used for the investigation of the effect of influenza viruses on this cell type [see e.g. 6], [37], [38]. If not otherwise stated, glycyrrhizin was continuously present in cell culture media starting with a 1 h pre-infection period. Glycyrrhizin 200 µg/ml (the maximum tested concentration) did not affect A549 cell viability (data not shown) but clearly decreased CPE formation in A549 cells infected with the H5N1 influenza strain A/Thailand/1(Kan-1)/04 at MOIs of 0.01, 0.1 or 1 (Figure 1A). Similar results were obtained in A549 cells infected with strain A/Vietnam/1203/04 (H5N1) (Suppl. Figure 1A). Staining of A549 cells for influenza A nucleoprotein 24 h after infection with strain H5N1 A/Thailand/1(Kan-1)/04 indicated that glycyrrhizin 200 µg/ml significantly reduces the number of influenza A nucleoprotein positive cells (Figure 1B).

To examine the influence of glycyrrhizin on virus progeny, A549 cells were infected with the H5N1 influenza strain A/Thailand/1(Kan-1)/04 at MOI 0.01 or MOI 1 and infectious virus titres were determined 24 h post infection (Figure 1C). While glycyrrhizin in concentrations up to 50 µg/ml did not affect H5N1 replication, moderate effects were exerted by glycyrrhizin 100 µg/ml and more pronounced effects by glycyrrhizin 200 µg/ml (MOI 0.01∶ 13-fold reduction, MOI 1∶ 10-fold reduction). Next, influence of glycyrrhizin on H5N1 replication was confirmed by the detection of viral (H5) RNA using quantitative PCR. Only glycyrrhizin concentrations ≥100 µg/ml significantly reduced viral RNA expression in H5N1 A/Thailand/1(Kan-1)/04-infected (Suppl. Figure 1B) or H5N1 A/Vietnam/1203/04-infected (Suppl. Figure 1C) A549 cells (MOI 0.01) 24 h post infection.

Time-of-addition experiments revealed that maximal effects were achieved when glycyrrhizin was continuously present starting with a 1 h pre-incubation period (Figure 1D). Addition of glycyrrhizin post infection showed reduced antiviral effects while pre-incubation alone or glycyrrhizin addition during the adsorption period did not significantly affect H5N1 replication.

### Influence of glycyrrhizin on cytokine production in A549 cells

**Figure 2:**
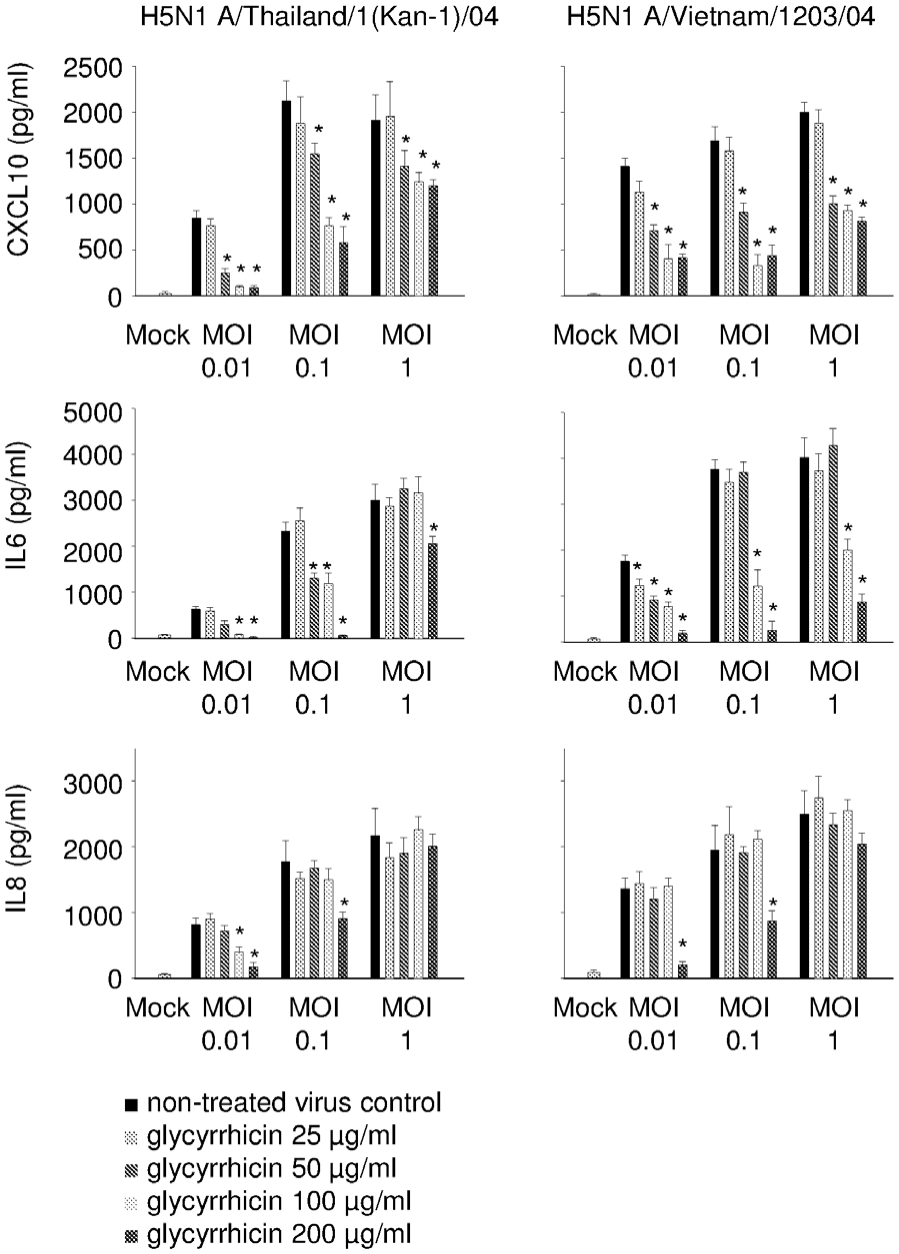
Influence of glycyrrhizin on H5N1-induced expression of CXCL10, IL6, and IL8 in A549 cells. Excretion of cytokines by non-infected (Mock) A549 cells or A549 cells infected with different H5N1 strains at different MOIs 24 h post infection determined by ELISA. For each MOI five bars are presented. From the left to the right these bars represent: untreated virus control, glycyrrhizin 25 µg/ml, glycyrrhizin 50 µg/ml, glycyrrhizin 100 µg/ml, glycyrrhizin 200 µg/ml. * P<0.05 relative to non-treated virus control.

**Figure 3:**
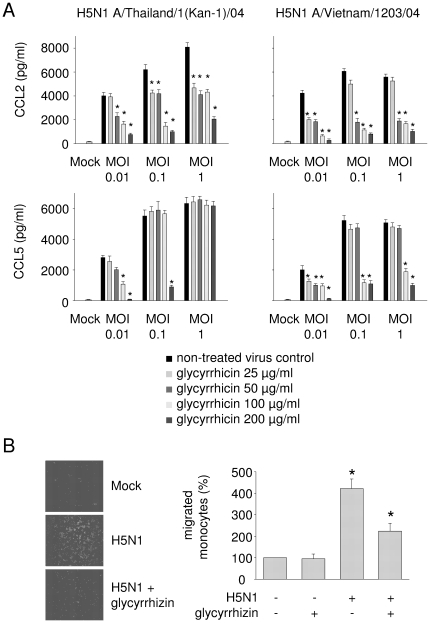
Influence of glycyrrhizin on H5N1-induced expression of CCL2 and CCL5 in A549 cells and on monocyte migration towards supernatants of H5N1-infected A549 cells. A) Excretion of cytokines by non-infected (Mock) A549 cells or A549 cells infected with different H5N1 strains at different MOIs 24 h post infection determined by ELISA. For each MOI five bars are presented. From the left to the right these bars represent: untreated virus control, glycyrrhizin 25 µg/ml, glycyrrhizin 50 µg/ml, glycyrrhizin 100 µg/ml, glycyrrhizin 200 µg/ml. * P<0.05 relative to non-treated virus control. B) Representative pictures of monocytes migrated towards supernatants of H5N1-infected A549 cells and quantification of migrated monocytes towards supernatants of H5N1-infected A549 cells relative to supernatants of non-treated Mock cells. Primary human monocytes (10^6^ cells) were seeded on 8 µm filters. The filters were placed into wells containing supernatants of non-infected (Mock) or H5N1 A/Thailand/1(Kan-1)/04 (MOI 0.1)-infected glycyrrhizin (100 µg/ml)-treated or non-treated A549 cells. After 24 h, the migrated monocytes were quantified after fixation and DAPI staining of the cells attached to the lower surface of the membranes. Five random fields (each 0.25 mm^2^) were counted at 200× magnification. * P<0.05 relative to supernatants of non-treated Mock cells.

For investigation of H5N1-induced cytokine expression, five pro-inflammatory genes were chosen that had been correlated to severity of influenza disease: CXCL10 (also known as interferon-γ-inducible protein 10, IP-10), interleukin 6 (IL6), interleukin 8, (IL8; also known as CXCL8), CCL2 (also known as monocyte chemoattractant protein 1, MCP-1), and CCL5 (also known as RANTES). A549 cells were infected with H5N1 A/Thailand/1(Kan-1)/04 or H5N1 A/Vietnam/1203/04 at MOI 0.01, 0.1, or 1. Glycyrrhizin treatment was performed with 25, 50, 100, or 200 µg/ml. Cytokine expression was detected 24 h post infection by ELISA. Glycyrrhizin did not affect cytokine expression of non-infected cells (data not shown) but inhibited expression of all cytokines investigated in H5N1-infected cells in a dose-dependent manner (Figure 2, Figure 3A). Effects were more pronounced at lower MOIs. Notably, expression of all cytokines except IL8 was significantly inhibited after treatment with glycyrrhizin 50 µg/ml or even 25 µg/ml at MOI 0.01 (Figure 2, Figure 3A) although these glycyrrhizin concentrations had no effect on H5N1 replication in A549 cells (Figure 1, Figure S1).

### Influence of glycyrrhizin on monocyte recruitment of H5N1-infected A549 cells

Cytokine expression by influenza A virus-infected respiratory cells causes recruitment of peripheral blood monocytes into the lungs of patients where they differentiate to macrophages which are thought to contribute to influenza A virus pathogenicity [5], [39]. In a chemotaxis assay, the influence of glycyrrhizin was investigated on migration of monocytes towards supernatants of H5N1 A/Thailand/1(Kan-1)/04 (MOI 0.1)-infected A549 cells through 8 µm filters. Monocyte migration towards supernatants of H5N1-infected cells was strongly increased relative to migration towards supernatants of non-infected cells. Treatment of H5N1-infected cells with glycyrrhizin 100 µg/ml clearly suppressed chemoattraction activity of supernatants (Figure 3B).

### Influence of glycyrrhizin on H5N1-induced caspase activation and nuclear export of ribonucleoprotein (RNP) complexes

**Figure 4:**
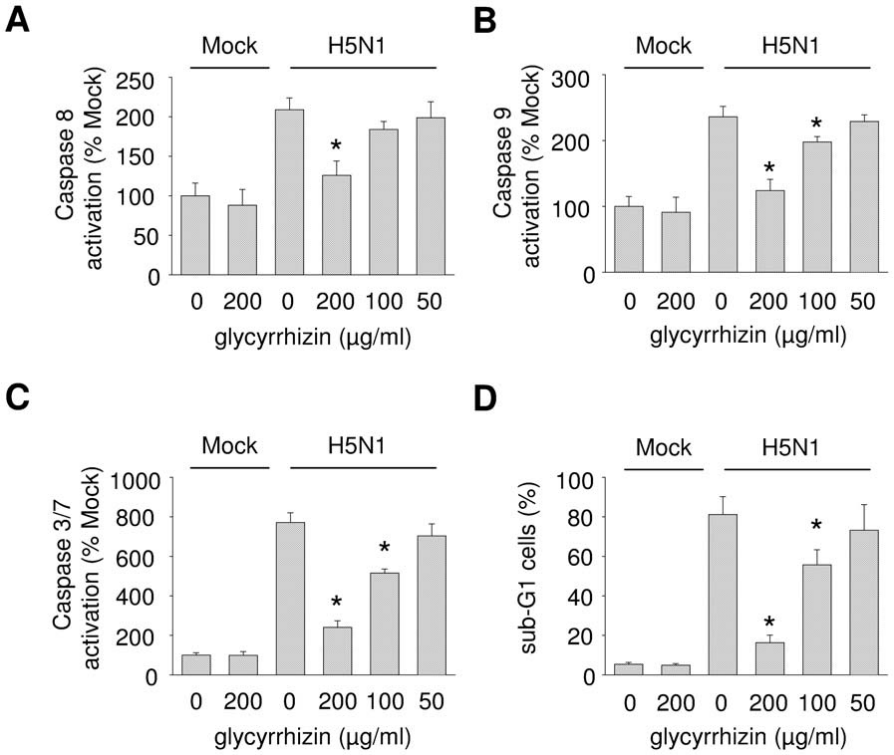
Influence of glycyrrhizin on H5N1-induced caspase activation and cells in sub-G1 phase. Caspase activation (A–C) or fraction of cells in sub-G1 phase (D) were measured in non-infected (Mock) or H5N1 A/Thailand/1(Kan-1)/04 (MOI 0.01)-infected glycyrrhizin-treated or non-treated A549 cells 24 h post infection.

Influenza viruses including H5N1 have been shown to induce caspase-dependent apoptosis in airway cells and this apoptosis has been correlated to the virus pathogenicity [40], [41]. Glycyrrhizin concentrations up to 200 µg/ml did not affect caspase activation in non-infected cells (Figure 4A–C). Glycyrrhizin concentrations ≥100 µg/ml inhibited H5N1 A/Thailand/1(Kan-1)/04 (MOI 0.01)-induced activation of the initiator caspases 8 and 9 as well as of the effector caspases 3/7 in A549 cells as determined 24 h post infection (Figure 4A–C). Lower glycyrrhizin concentrations did not affect H5N1-induced apoptosis. The detection of cells in sub-G1 phase resulted in similar findings (Figure 4D).

Substances that inhibit H5N1-induced caspase 3 activation including caspase 3 inhibitors cause nuclear retention of RNP complexes [34], [42]. In accordance, glycyrrhizin also interfered with nuclear export RNP at MOI 1 (Figure S2). Similar results were obtained in MOI 0.01 H5N1 A/Thailand/1(Kan-1)/04-infected cells (Figure S3).

### Influence of glycyrrhizin on H5N1-induced activation of nuclear factor κB (NFκB), p38, and on H5N1-induced cellular reactive oxygen species (ROS) formation

**Figure 5:**
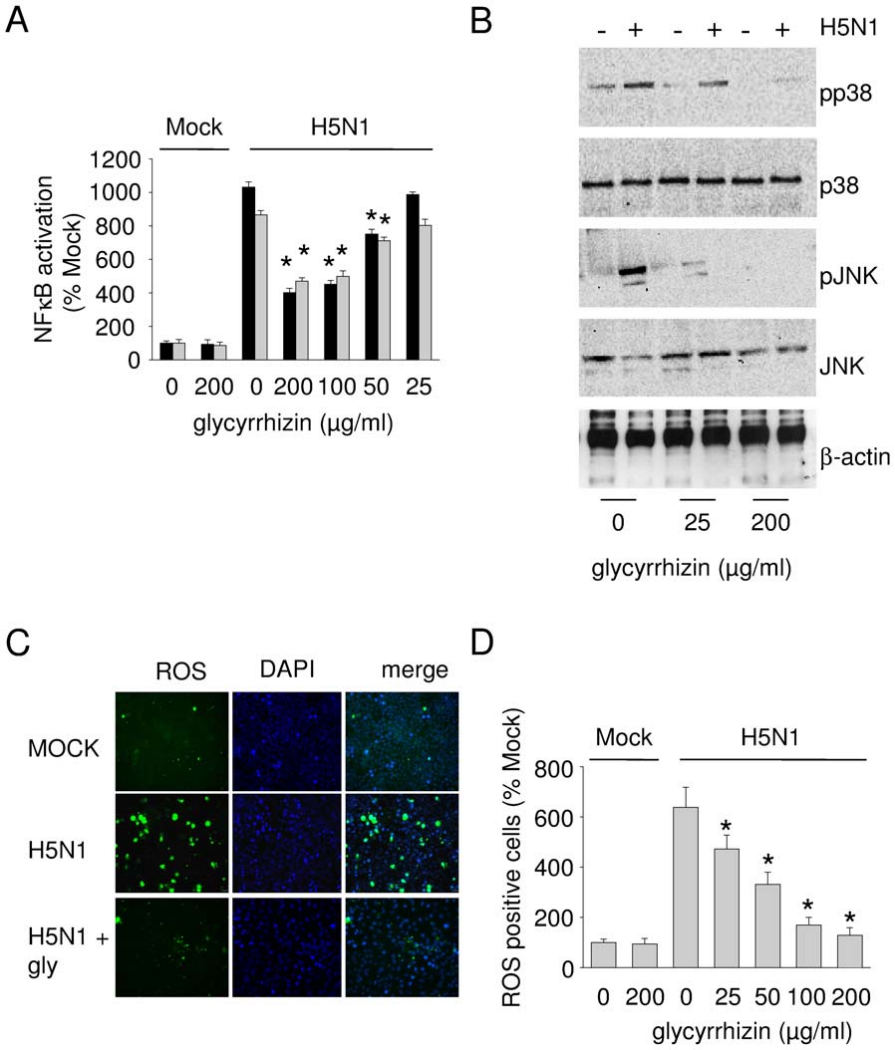
Influence of glycyrrhizin on activation of NFκB, p38, and on H5N1-induced formation of reactive oxygen species (ROS). A) NFκB activity examined by determination of the activities of the NFκB subunits p65 (black bars) and p50 (grey bars) in non-infected (MOCK) or H5N1 A/Thailand/1(Kan-1)/04 (MOI 0.01)-infected glycyrrhizin-treated or non-treated A549 cells 24 h post infection. * P<0.05 relative to non-treated virus control. B) Western blots showing levels of p38, phosphorylated p38 (pp38), JNK, phosphorylated JNK (pJNK), or β-actin (loading control) in non-infected or H5N1 A/Thailand/1(Kan-1)/04 (MOI 0.01)-infected glycyrrhizin-treated or non-treated A549 cells 1 h post infection. C) Representative pictures showing H5N1 A/Thailand/1(Kan-1)/04 (MOI 0.01)-induced ROS formation in the presence or absence of glycyrrhizine 100 µg/ml (gly) 24h post infection in A549 cells. ROS are indicated in green. Nuclei are DAPI-stained (blue). D) Quantification of ROS-positive cells with or without glycyrrhizin treatment. * P<0.05 relative to non-treated virus control.

Activation of NFκB, p38, and JNK have been associated with influenza A virus replication and virus-induced pro-inflammatory gene expression [34], [43]–[47]. While glycyrrhizin did not influence NFκB activity in non-infected A549 cells in the tested concentrations (data not shown), glycyrrhizin inhibited NFκB activation in H5N1-infected cells (Figure 5A). Moreover, glycyrrhizin inhibited H5N1-induced phosphorylation of the MAPKs p38 and JNK (Figure 5B).

In addition to their roles during influenza A virus replication and virus-induced cytokine/chemokine expression, NFκB, p38, and JNK are constituents of redox-sensitive signalling pathways [48]–[51]. Antioxidants had been already found to interfere with influenza A virus-induced signalling through NFκB, p38, and JNK, with influenza A virus replication, and with influenza A virus-induced pro-inflammatory gene expression [32]–[34]. Since glycyrrhizin is known to exert antioxidative effects [26] we speculated that glycyrrhizin may interfere with H5N1-induced ROS formation. Indeed glycyrrhizin exerted clear antioxidative effects in H5N1 (MOI 0.01)-infected cells (Figure 5C) causing significant reduction of ROS formation already at a concentration of 25 µg/ml (Figure 5D).

## Discussion

Here, we show that glycyrrhizin inhibits the replication of highly pathogenic H5N1 influenza A virus, H5N1-induced apoptosis, and H5N1-induced expression of pro-inflammatory cytokines in lung-derived A549 cells. After intravenous administration, achievable plasma concentrations of glycyrrhizin have been described to be about 100 µg/ml [52]. Therefore, the glycyrrhizin concentrations found to interfere with H5N1 replication and H5N1-induced pro-inflammatory gene expression in the present report are in the range of therapeutic plasma levels. Notably, although higher glycyrrhizin concentrations were needed to interfere with SARS coronavirus replication [22] than with H5N1 replication, beneficial results were reported in glycyrrhizin (SNMC)-treated SARS patients in comparison to SARS patients who did not receive glycyrrhizin [23]. Notably, investigation of different glycyrrhizin derivatives against SARS coronavirus led to the identification of compounds with enhanced antiviral activity [53]. Therefore, glycyrrhizin might also serve as lead structure for the development of novel anti-influenza drugs.

Experimental results suggested that glycyrrhizin might be able to affect seasonal influenza A virus disease by antiviral and immunomodulatory effects [26], [27]. Mice were prevented from lethal H2N2 infection by glycyrrhizin although no influence on virus replication was detected. The mechanism was suggested to be induction of interferon-γ in T-cells by glycyrrhizin [54]. Moreover, glycyrrhizin was shown to influence seasonal influenza A virus replication through interaction with the cell membrane [25], [28]. However, these effects were observed only in concentrations ≥200 µg/ml when glycyrrhizin was added during the virus adsorption period. Since glycyrrhizin addition during the adsorption period did not influence H5N1 replication in our experiments it appears not likely that membrane effects contribute to anti-H5N1 effects detected here in lower concentrations.

Our results rather suggest that glycyrrhizin interferes with H5N1-induced oxidative stress. Influenza A virus (including H5N1) infection induces ROS formation. Antioxidants were found to inhibit influenza A virus replication and influenza A virus-induced pro-inflammatory gene expression [32]–[34] and glycyrrhizin is known to exert antioxidative effects [26]. Here, glycyrrhizin interfered with H5N1-induced activation of NFκB, p38, and JNK representing redox-sensitive signalling events [48]–[51] involved in influenza A virus replication and influenza A virus-induced cellular cytokine/chemokine production [34], [43]–[46], [55]. Glycyrrhizin 50 µg/ml significantly reduced H5N1-induced activation of NFκB. In addition, glycyrrhizin concentrations as low as 25 µg/ml effectively interfered with H5N1-induced ROS formation and with phosphorylation of the redox-sensitive MAPKs p38 and JNK. In our model, activation of p38 appears to be critical for H5N1-associated redox signalling since p38 inhibition had been shown before to mimick effects of the antioxidant N-acetyl-cysteine (NAC) [34]. Interestingly and in contrast to glycyrrhizin, NAC failed to inhibit H5N1 replication or H5N1-induced cytokine/chemokine expression in therapeutically relevant concentrations.

Glycyrrhizin diminished H5N1-induced cellular cytokine/chemokine production in concentrations (≤50 µg/ml) that did not interfere with H5N1 replication although redox-sensitive signalling pathways have been described to be involved in both processes. Therefore, H5N1-induced proinflammatory gene expression appears to be more sensitive to inhibition of ROS formation than H5N1 replication. Indeed, influenza viruses had been shown to induce cellular pathways through replication-dependent and –independent events [56]. In a previous report, we could show that similar glycyrrhizin concentrations like those investigated here interfered with H5N1-induced pro-inflammatory gene expression but not with H5N1 replication in human monocyte-derived macrophages [57]. In addition, other immunomodulatory treatment regimens that did not influence H5N1 replication reduced mortality in H5N1-infected mice [31], [58]. Therefore, glycyrrhizin represents a potential additional treatment option that interfers with both H5N1 replication and H5N1-induced expression of pro-inflammatory cytokines in lung cells.

Interference with immune responses may also result in the loss of control of virus replication by cytotoxic immune cells including natural killer cells and cytotoxic CD8^+^ T-lymphocytes. Global immunosuppressants like corticosteroids failed to protect from lethal influenza virus infection [59]. Moreover, antiviral drugs may interfere with cytotoxic cells that control virus replication as demonstrated for ribavirin that was shown to hamper NK cell cytolytic activity [60]. In this context, glycyrrhizin had already been shown not to affect natural killer cell activity in the concentrations used here [57].

In conclusion, we show in this report that therapeutic concentrations of glycyrrhizin (used as clinically approved parenteral preparation SNMC) interfere with highly pathogenic H5N1 influenza A virus replication and H5N1-induced pro-inflammatory gene expression at least in part through interference with H5N1-induced ROS formation and in turn reduced activation of p38, JNK, and NFκB in lung cells. Since we used the clinical formulation SNMC effects of other ingredients like glycin or cystein cannot be excluded. Vaccines and antiviral agents will fail to meet global needs at least at the beginning of a severe influenza A virus pandemic [61]. Anti-inflammatory and immunomodulatory agents are considered to be important candidates as constituents of anti-influenza treatment strategies that may save lives in an influenza pandemic situation [61]. Therefore, glycyrrhizin may complement the arsenal of potential drugs for the treatment of H5N1-caused disease.

## Supporting Information

Figure S1 **Influence of glycyrrhizin on H5N1 replication in A549 cells.** A) Representative pictures of non-infected A549 cells (Mock) and cytopathogenic effect formation in A549 cells infected with H5N1 strain A/Vietnam/1203/04 at different multiplicities of infection (MOI) without or with glycyrrhizin (200 µg/ml) treatment for 24 h. B) and C) Effect of different glycyrrhizin concentrations on expression of influenza RNA detected by quantitative PCR in H5N1 A/Thailand/1(Kan-1)/04-infected (B) or A/Vietnam/1203/04-infected (C) A549 cells (MOI 0.01) 24 h post infection. * P<0.05 relative to non-treated control.
(PDF)

Figure S2 **Influence of glycyrrhizin on nuclear export of influenza A virus ribonucleoprotein (RNP) complexes.** Influence of glycyrrhizine (Gly) on nuclear export of viral NP indicating RNP complexes in H5N1 A/Thailand/1(Kan-1)/04 (MOI 1)-infected A549 cells 8 h p.i. RNP localisation (green) was visualised by fluorescence microscopy using an antibody directed against influenza A NP. Nuclei are stained by DAPI (shown in blue).
(PDF)

Figure S3 **Influence of glycyrrhizin on nuclear export of influenza A virus ribonucleoprotein (RNP) complexes.** Influence of glycyrrhizine (Gly) on nuclear export of viral NP indicating RNP complexes in H5N1 A/Thailand/1(Kan-1)/04 (MOI 0.01)-infected A549 cells 8 h p.i. RNP localisation (green) was visualised by fluorescence microscopy using an antibody directed against influenza A NP. Nuclei are stained by DAPI (shown in blue).
(PDF)
