# Supplementary material for: Glycyrrhizin Exerts Antioxidative Effects in H5N1 Influenza A Virus-Infected Cells and Inhibits Virus Replication and Pro-Inflammatory Gene Expression
Source: PLoS One. 2011 May 17;6(5):e19705. doi: 10.1371/journal.pone.0019705 (PMC3096629; doi:10.1371/journal.pone.0019705)
Supplement: Figure S1 — Influence of glycyrrhizin on H5N1 replication in A549 cells. A) Representative pictures of non-infected A549 cells (Mock) and cytopathogenic effect formation in A549 cells infected with H5N1 strain A/Vietnam/1203/04 at different multiplicities of infection (MOI) without or with glycyrrhizin (200 µg/ml) treatment for 24 h. B) and C) Effect of different glycyrrhizin concentrations on expression of influenza RNA detected by quantitative PCR in H5N1 A/Thailand/1(Kan-1)/04-infected (B) or A/Vietnam/1203/04-infected (C) A549 cells (MOI 0.01) 24 h post infection. * P<0.05 relative to non-treated control. (PDF) [file pone.0019705.s001.pdf]

Figure S1

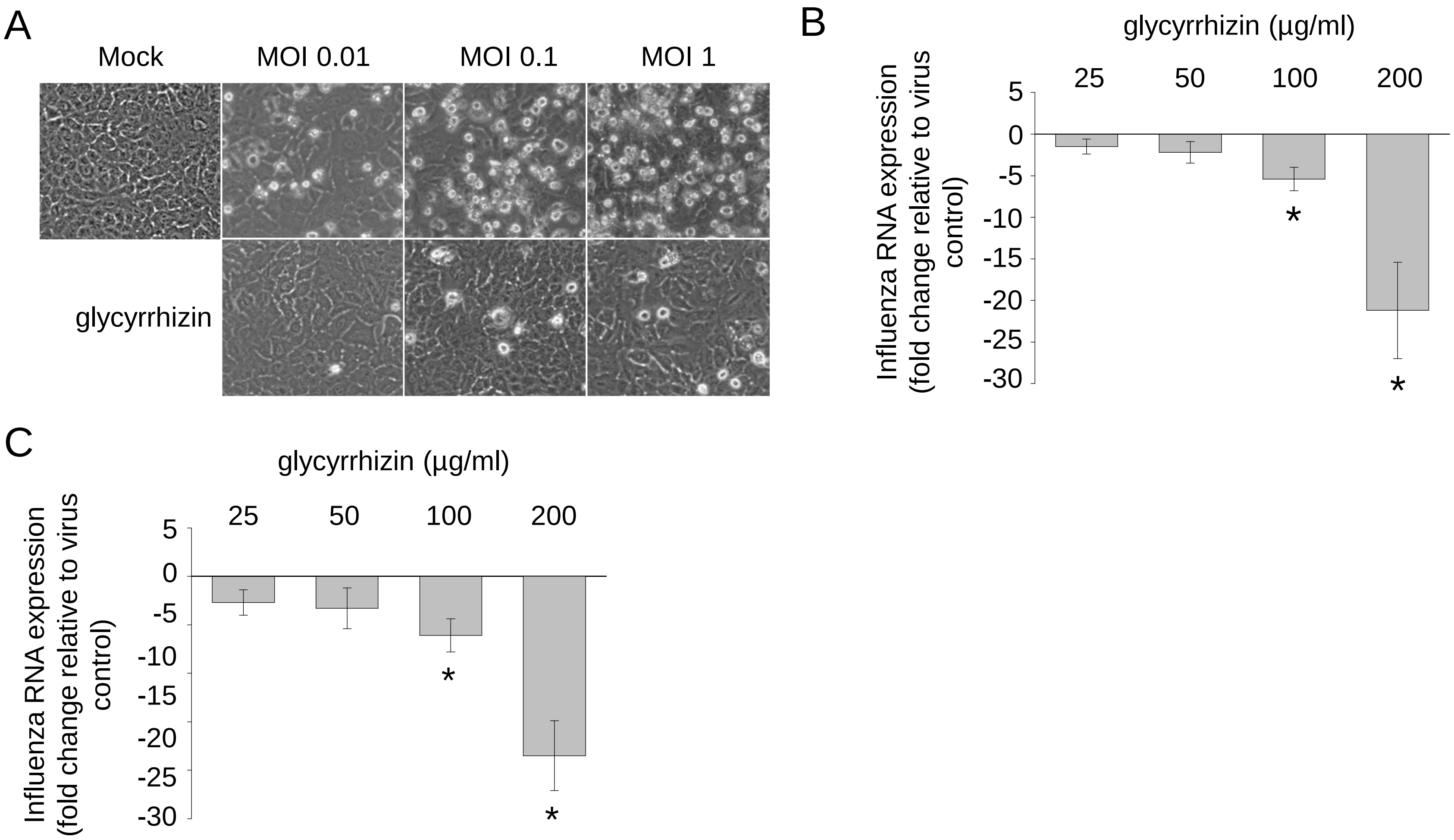

Figure S1. Influence of glycyrrhizin on H5N1 replication in A549 cells. A) Representative pictures of non-infected A549 cells (Mock) and cytopathogenic effect formation in A549 cells infected with H5N1 strain A/Vietnam/1203/04 at different multiplicities of infection (MOI) without or with glycyrrhizin (200 µg/ml) treatment for 24 h. B) and C) Effect of different glycyrrhizin concentrations on expression of influenza RNA detected by quantitative PCR in H5N1 A/Thailand/1(Kan-1)/04-infected (B) or A/Vietnam/1203/04-infected (C) A549 cells (MOI 0.01) 24 h post infection. * $P < 0.05$ relative to non-treated control
