# Supplementary material for: Glycyrrhizin Exerts Antioxidative Effects in H5N1 Influenza A Virus-Infected Cells and Inhibits Virus Replication and Pro-Inflammatory Gene Expression
Source: PLoS One. 2011 May 17;6(5):e19705. doi: 10.1371/journal.pone.0019705 (PMC3096629; doi:10.1371/journal.pone.0019705)
Supplement: Figure S3 — Influence of glycyrrhizin on nuclear export of influenza A virus ribonucleoprotein (RNP) complexes. Influence of glycyrrhizine (Gly) on nuclear export of viral NP indicating RNP complexes in H5N1 A/Thailand/1(Kan-1)/04 (MOI 0.01)-infected A549 cells 8 h p.i. RNP localisation (green) was visualised by fluorescence microscopy using an antibody directed against influenza A NP. Nuclei are stained by DAPI (shown in blue). (PDF) [file pone.0019705.s003.pdf]

Figure S3

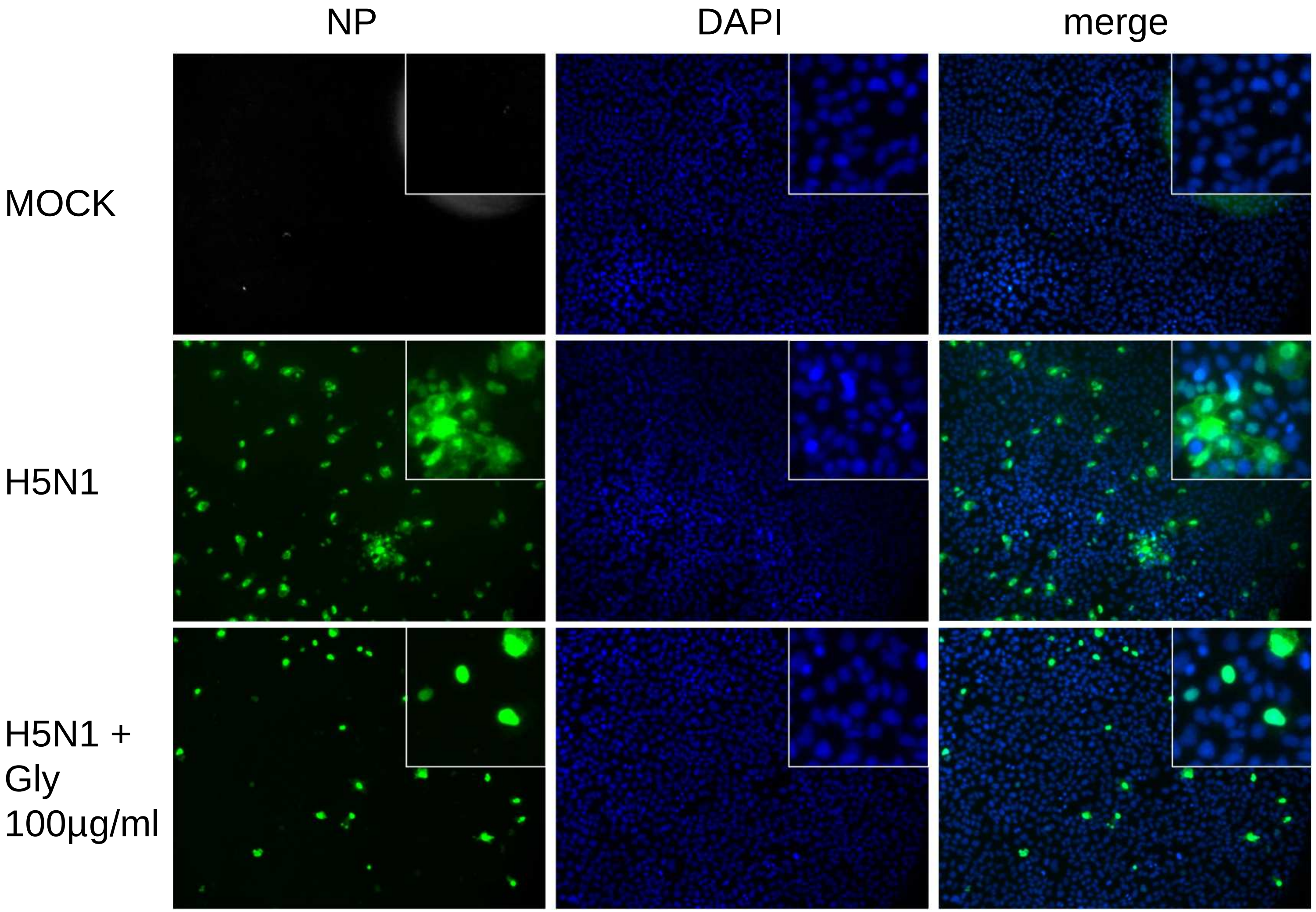

Figure S3. **Influence of glycyrrhizin on nuclear export of influenza A virus ribonucleoprotein (RNP) complexes.** Influence of glycyrrhizine (Gly) on nuclear export of viral NP indicating RNP complexes in H5N1 A/Thailand/1(Kan-1)/04 (MOI 0.01)-infected A549 cells 8h p.i. RNP localisation (green) was visualised by fluorescence microscopy using an antibody directed against influenza A NP. Nuclei are stained by DAPI (shown in blue).
